# Role of telemedicine in bridging the healthcare gap: application of the hub and spokes model in the Hohoe Municipality, Ghana

**DOI:** 10.3389/fdgth.2026.1864794

**Published:** 2026-08-12

**Authors:** Bridget Setornyo Letsa, Joshua Oppong, Habibu Ahmed, Chrysantus Kubio, Anthony Ofosu Adofo, Hubert Amu, Frank Baiden

**Affiliations:** 1 Volta Regional Hospital, Ghana Health Service, Hohoe, Ghana; 2 Department of Population and Behavioral Sciences, Fred N. Binka School of Public Health, University of Health and Allied Sciences, Hohoe, Ghana; 3 Northern Regional Health Directorate, Ghana Health Service, Tamale, Ghana; 4 Department of Community Health, School of Medicine, University of Health and Allied Sciences, Ho, Ghana; 5 Department of Epidemiology and Biostatistics, Fred N. Binka School of Public Health, University of Health and Allied Sciences, Hohoe, Ghana

**Keywords:** case management, health services accessibility, maternal health, quality of health care, referral and consultation, telemedicine

## Abstract

**Background:**

Despite ongoing efforts toward achieving Universal Health Coverage in Ghana, access to healthcare in rural areas is still limited. Among the services that are accessible, there is still a gap in the quality of the services provided in rural areas. Telemedicine offers a promising solution, but evidence of its role and effectiveness is yet to be fully realized. This study, therefore, explored the role of telemedicine in bridging the healthcare gap in the Hohoe Municipality.

**Methods:**

A mixed-methods approach was employed, combining in-depth interviews with 23 health professionals and 16 patients. A retrospective chart review of telemedicine records from October 2021 to October 2023 was conducted. Descriptive analysis was performed on the quantitative data with Stata V.17, while qualitative data collected were analyzed thematically using Atlas.ti v7.5.

**Results:**

Out of 1,411 patients who used telemedicine services, 40% of the conditions were maternal and 89.1% of the consultations were referred to a higher facility with only 2.2% resulting in aborted referrals. Telemedicine plays a role in terms of accessibility (bridging geographical barriers), smoothing referrals, case management, and improving service delivery. Patients were highly satisfied with the telemedicine services. However, challenges faced included unstable network connectivity, unavailability of telephones (which made providers use their personal mobile phones), and other logistical challenges.

**Conclusion:**

Our findings highlight the role of telemedicine in bridging the healthcare access gap and its contribution to the healthcare system through the Hub and Spoke model in the Hohoe Municipality. To accelerate progress towards attainment of the Sustainable Development Goal (SDG) target 3.8 on universal health coverage, we recommend that telemedicine be extended to all health facilities in Ghana, especially the rural areas, while addressing the challenges inherent in its implementation.

## Introduction

Globally, 50% of the population is unable to access primary healthcare services despite healthcare accessibility being a fundamental human right (1) and 800 million people spend over 10% of their income on seeking healthcare services (2, 3). In Sub-Saharan Africa (SSA), healthcare accessibility remains challenged in most parts of the continent, with only 42.6% of people having access to healthcare, which has increased the mortality and morbidity rates of diseases (4). In Ghana, more than half of the population (61%) has access to primary health care. However, utilization and the quality of care provided are still problematic, particularly in remote locations (5). Geographical barriers and financial constraints are the most common barriers influencing the accessibility of quality healthcare services (6, 7).

Telemedicine was introduced to address the gaps in geographical, financial, sociocultural, and infrastructural barriers in the provision of healthcare services (8, 9). Telemedicine is a technology that uses online platforms to provide healthcare services (10). It connects health professionals across the healthcare system in a single unit of health services delivery. The integration of telemedicine into healthcare systems has been effective in overcoming geographical obstacles and enhancing healthcare accessibility (11, 12) and is now more practical due to technological improvements and increased accessibility to information technology (13).

Telemedicine adoption and impact differ between urban and rural settings. Urban and metropolitan areas typically benefit from stronger technological infrastructure and more reliable internet connectivity, which have supported wider telemedicine uptake (14). In rural areas, particularly in low- and middle-income countries, poor or unstable internet connectivity and limited access to equipment remain significant barriers to adoption (15), even though rural populations often stand to benefit the most, given greater distances to specialist care and fewer locally available health workers. This urban-rural disparity demonstrates the need for context-specific models, such as the Hub and Spokes system examined in this study.

The achievement of universal health coverage (UHC) is a major goal of the Sustainable Development Goals (SDGs), which are aimed to be achieved worldwide by 2030. This highlights the importance of programs that improve accessibility to healthcare (2). Achieving SDG target 3.8 will reduce the rate of morbidity and mortality and improve quality of life (2, 16). Telemedicine holds the promise of contributing to this goal.

Rural communities in Ghana continue to face significant inequities in healthcare access due to geographical barriers, infrastructural constraints, and a lack of skilled personnel (17), despite Telemedicine's potential to address these issues if implemented widely. Exploring how telemedicine functions in Ghana and facilitates access to quality care is critical for informing policies aimed at achieving SDG 3.8 on UHC. While telemedicine has been piloted in various forms across Ghana and other LMICs, there remains limited evidence that combines routinely collected service data with the experiences of both providers and patients in a single Hub-and-Spokes system operating in rural practice. Therefore, this study investigates how telemedicine, structured through a hub-and-spokes model, addresses healthcare accessibility gaps in the Hohoe Municipality of Ghana.

## Materials and methods

Our study adopted the guidelines outlined in STROBE (quantitative strand), COREQ (qualitative strand), and GRAMMS (mixed-methods integration and reporting).

### Study site

**Figure 1:**
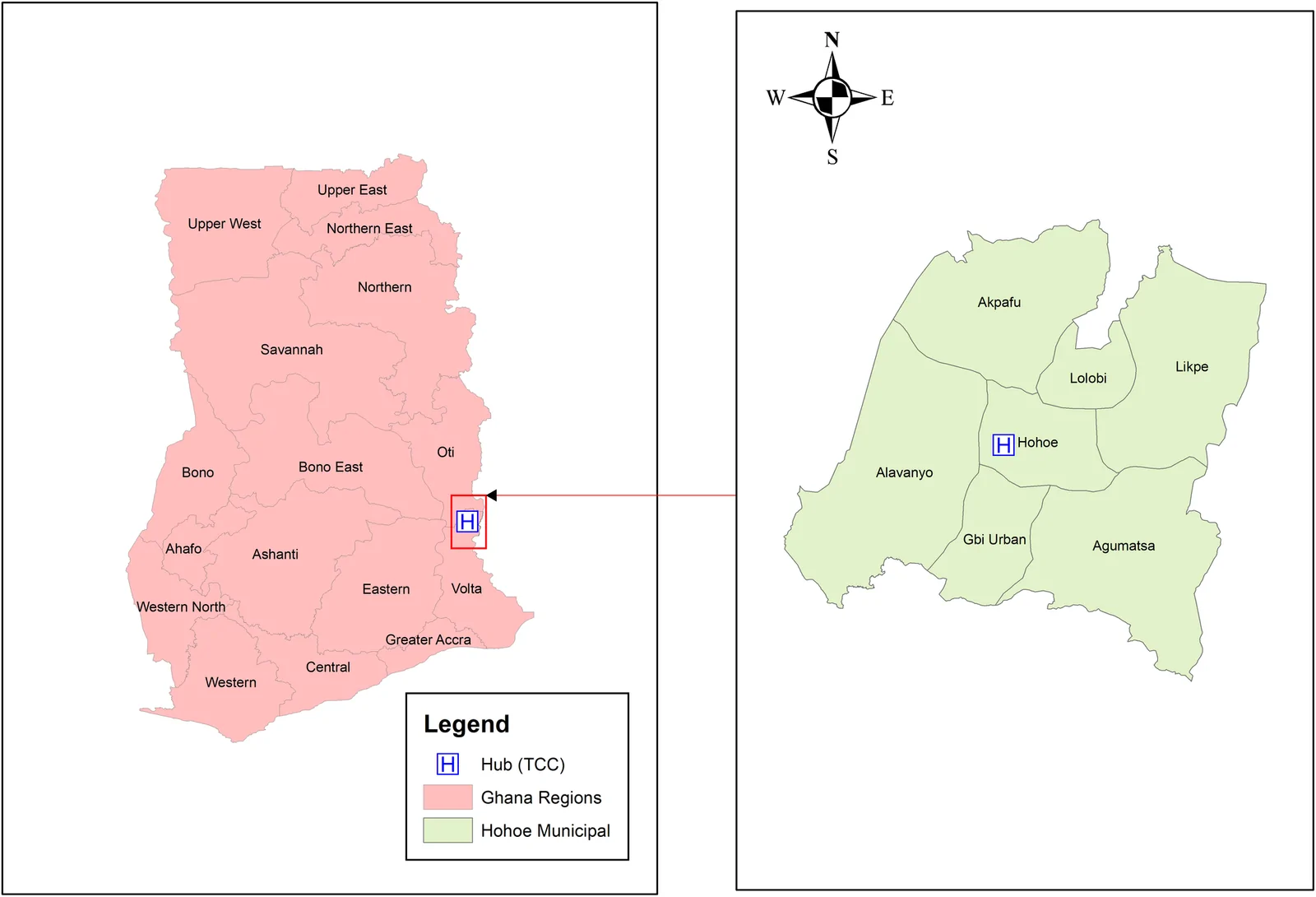
Map of Ghana showing the location of hohoe municipality and the Teleconsultation Centre (TCC) hub.

Our study was conducted in health facilities using telemedicine in the Hohoe Municipality of the Volta Region of Ghana, as shown in Figure 1. The Municipality is located between longitude 0° 15’E and 0° 45’E and latitude 6° 45’N and 7° 15’N. The Municipality shares borders with the Republic of Togo on the east, on the southeast with Afadzato South District, and on the southwest with Kpando Municipality, both in the Volta Region; on the north with the Guan District; and on the northwest with the Biakoye District, both in the Oti Region. Hohoe District was created in 1979 after being carved out of the ‘old’ Jasikan and Kpando District Councils and attained its municipal status in 2008. Its capital, Hohoe, is located about 78 kilometers away from Ho, the regional capital. The Municipality consists of one hundred and two (102) communities with a population of 114,472 (18). The Municipality has several CHPS compounds, Health centers, and a hospital that serves as a regional hospital.

Telemedicine in Ghana uses information and communications technology (ICT) to bridge gaps in healthcare delivery, especially for rural and underserved populations. A key component of this approach is the Teleconsultation Centre (TCC), a facility staffed with trained health professionals who provide clinical advice and support remotely to community health workers and patients. In the Hohoe municipality, TCC serves four main sub-districts: Alavanyo Wudidi, Agumatsa, Gbi urban, and Hohoe, and it was established in 2021.

### Study design

Our study employed a concurrent mixed-method design (qualitative and quantitative methods) to explore the role of telemedicine in bridging the healthcare gap. The design was chosen to expand evidence, improve the credibility of the study findings, and illustrate results from one method with the results of the other (19). The quantitative aspect of the study was a retrospective-descriptive cross-sectional design, while the qualitative aspect employed an exploratory-descriptive design to gather in-depth information on the role of telemedicine and challenges from health professionals’ and patients’ perspectives. The retrospective chart review was conducted using telemedicine records documented in the telemedicine encounter book at the TCC. The review aimed to assess patterns of telemedicine use, referral outcomes, and disease categories managed through teleconsultation between October 2021 and October 2023. To ensure data completeness and relevance, only teleconsultation records that contained complete information on patient age, sex, clinical condition, and teleconsultation outcome were included in the analysis.

### Conceptual framework

According to the Hub and Spokes concept (HSM) for health service delivery, basic services are supplied at secondary locations while essential resources are centralized at a single location where specialized services may be offered (20). The model's layout produces a healthcare network with a better-resourced main site that serves outlying sites and offers essential basic services. Most of the implementation of telemedicine systems utilized the HSM, with hospitals serving as the hub and other facilities, like assisted living facilities, serving as the spokes (21).

The idea of the model was pioneered in the airport industry in 1952, but it was first introduced by Dr. Bertrand Dawson in 1920 in healthcare (22). HSM is more likely to be advantageous in developing countries where diseases are becoming more prevalent as a result of urbanization and changing demographics. Since poor infrastructure is a major issue in these areas, the spokes can benefit from the knowledge and highly skilled labor of the rich in resource hubs to deliver improved services (20).

In the Hohoe Municipality, the HSM links 14 lower-level healthcare facilities (spokes) to the central Teleconsultation Centre (TCC) at the Volta Regional Hospital (VRH), which serves as the hub. The hub is staffed with specialists, doctors, midwives, and well-trained nurses, and is equipped with the resources needed to support the spokes. Through this model, health professionals at peripheral facilities contact the TCC primarily by phone for guidance and support in managing cases that exceed their local capacity.

The hub is expected to provide timely advice, ensure continuity of care, and facilitate referrals only when necessary, thereby reducing avoidable transfers. Services delivered through this platform include obstetric and maternal care, management of acute conditions, and general case consultations. Importantly, the model also enhances the skills of health personnel at the spokes, who gain new experiences and confidence through ongoing interaction with specialists at the hub while expanding quality care to patients.

### Study population

Our study was conducted among healthcare professionals within the Hohoe Municipality who were actively involved in running telemedicine, along with patients who had experienced service delivery through telemedicine. The study also included only medical records in the telemedicine encounter book dated from October 2021 to October 2023. Our study excluded all telemedicine staff who were on annual leave, study leave, or not on duty during the period of data collection. Incomplete medical records were also excluded from the study.

### Sample size determination

For the quantitative data, a census of all client records from October 2021 to October 2023 was used. A total of 1,411 encounters were extracted. Hence, no formal sample size calculation was required.

For the qualitative data, 23 health professionals, two (2) from the hub, twenty-one (21) from the spokes, and 16 patients were recruited based on thematic saturation, a point at which no new codes or themes emerged from successive interviews. Data saturation was assessed iteratively throughout the study. Preliminary coding and theme development were conducted concurrently with data collection, and participant recruitment continued until no new codes or themes emerged from successive interviews. None of the patients refused participation. This approach ensured adequate coverage of perspectives relevant to the study objectives.

### Sampling

#### Qualitative sampling

Purposive sampling procedures were used to select health professionals. The participants were purposively selected to participate in the study based on characteristics that were of interest; thus, health professionals with different cadres (nurses, physician assistants, midwives) were selected in our study. These health professionals were sampled because of their active involvement in the day-to-day delivery of healthcare services through TCC. Patients/clients were also conveniently selected due to their availability.

#### Data collection procedure and instrument

A records review form (see Supplementary file) served as the instrument for collecting patients’ records from the telemedicine encounter books. The form was designed as a structured template with fields for relevant information such as age, sex, date of encounter, condition, and outcome. The record review form was deployed in Kobo Toolbox, which provided a simple interface that facilitated data entry, aiding in the systematic collection of data, simplifying management, and ensuring the validity of the data. Data were extracted by three health professionals (nurses) at the TCC to distribute the workload of manual entry.

The qualitative data were collected through an in-depth interview guide that served as the data collection instrument to gather in-depth information from participants. Two (2) interview guides were used (one for the patient and one for the healthcare professionals). The health professionals’ interview guide was structured into three sections (see Supplementary file). Section A: Socio-demographic characteristics, Section B: Role of telemedicine, Section C: Challenges of telemedicine. The patient interview guide was structured into two (see Supplementary file). Section A: Socio-demographic and Section B: Patient satisfaction with telemedicine services. An in-depth interview guide was adopted to enhance the investigation of experiences and views on the role of telemedicine in bridging the healthcare gap. All interviews with the health professionals were administered face-to-face and were conducted in English.

Patient interviews were conducted in the local dialect (Ewe), which was then transcribed into English during the data analysis. Patients were contacted after they had received care through the telemedicine service. Interviews were conducted face-to-face at the facility. Where the facility was too far, interviews were conducted by phone instead. The duration of the interviews ranged from 20 to 30 min for both patients and health professionals. Interviews were audio-recorded and field notes were also taken to avoid bias after which the recordings were transcribed verbatim. Both methods were used to ensure that interviews were not halted due to any malfunction of the recording device. There was no repetition of interviews. The interviews were led by the PI (BSL), who was a Master of Public Health student at the time of data collection, and three (3) research assistants. To ensure the trustworthiness of our findings after each interview, the field notes taken were referred to during the analysis, which included the participants’ body language. Also, our interview guides were reviewed by experts in the field of qualitative study to ensure the questions reflect the study objectives (23). Both the qualitative interviews and the quantitative chart review were conducted concurrently, from August to September 2024.

### Trustworthiness

To ensure trustworthiness, we followed Lincoln and Guba (1985) criteria: (1) credibility, (2) dependability, (3) transferability, and (4) confirmability (24). Credibility was ensured through the development of rapport with both healthcare professionals and patients before data collection. This created a safe environment that encouraged the health professionals and patients to share honest information about their experiences with telemedicine. Additionally, the interview guide was reviewed by two experts in qualitative research to ensure the instrument aligns with the study objectives and to enhance the quality of data collected. Dependability was ensured by involving experienced qualitative researchers in the study design, data collection, and analysis processes. We subjected the analysis process (transcription, coding, and theme development) to peer debriefing, whereby emerging themes were discussed with the research team throughout analysis to check consistency and minimize individual bias. We also ensured transferability by providing a detailed description of the study context, participant characteristics, and data collection procedures. This allows for replication of the study in similar healthcare settings, particularly in other rural or underserved communities. Finally, to ensure confirmability, ten health professionals and five patients reviewed the transcript and the results. This was to ensure that the interpretation and findings were based on participants’ experiences.

### Data analysis

The quantitative data were exported into Excel for data cleaning and further imported into STATA v17 for analysis. To ensure the quality of the data entered, a double entry was done to address discrepancies during data entry. The quantitative data were analyzed using descriptive analysis. Frequencies, graphs, and percentages were used to determine the current practices and standards, presenting patterns and trends in a clear and comprehensible manner.

For the qualitative analysis, all the recordings were transcribed verbatim and compiled into two Microsoft Word files, one for patients and the other for health professionals. To minimize errors, interview transcripts were compared with the interview notes and were proofread while listening to the audio recordings. Reflective thematic analysis was used to analyze the data following Braun and Clarke's (25) approach. The transcripts were read several times to ensure familiarity with the data and to identify key ideas and recurrent themes. Atlas.ti v7.5 was used to develop themes and codes, which were analytic and emergent in nature. Sentences, phrases, and words of relevance were coded. Similar codes were grouped to form subthemes, which were then organized into broader themes. The themes were presented in a manner that reflected the participants’ actual experiences. Direct quotes from participants were included to illustrate the findings and support key issues discussed. A frequency table was used to present the socio-demographic characteristics of the study participants. Quantitative and qualitative findings were merged during interpretation using a convergent (side-by-side) comparison, examining where qualitative themes converged with, diverged from, or expanded upon the quantitative results. This integrated interpretation (meta-inference) is presented in Table 5.

## Ethical considerations

Ethical approval was obtained from the University of Health and Allied Sciences (UHAS) Research Ethics Committee (REC) and protocol identification number UHAS-REC A.8[77] 23–24 was issued. Permission was also granted by the Volta Regional Hospital and Hohoe Municipal Health Directorate. The purpose of the study was introduced to the participants. Written informed consent was obtained from all participants involved before the commencement of the study. Unique codes were assigned to participants to anonymize their identity. Audio recordings and notes taken were kept on a password-protected device. Our study followed the ethical principles outlined in the Helsinki Declaration in human studies.

## Results

Table 1 presents the demographic profile and disease categories of Teleconsultation Records. The largest proportion (27.4%) were aged between 20 and 29 years and most of the encounters were female (76.8%). The majority of cases managed through teleconsultation were related to maternal health (40.0%), followed by Non-Communicable Diseases (24.9%). Encounters increased from 581 encounters (41.2% of the two-year total) in the first year to 830 encounters (58.8%) in the second year.

**Table 1:** Demographic profile and disease categories of teleconsultation records (*n* = 1,411).

Variable	Frequency (*n* = 1,411)	Percentage (%)
Age		
0–9	160	11.3
10–19	144	10.2
20–29	387	27.4
30–39	356	25.2
40–49	134	9.5
50–59	69	4.9
60–69	70	5.0
70–79	46	3.3
80–89	38	2.7
90–99	7	0.5
**Sex**		
Male	328	23.2
Female	1,083	76.8
Health Condition		
Abdominal Condition	38	2.7
Child Health	24	1.7
Gastrointestinal	33	2.3
Gynaecological	69	4.9
Infectious Disease	75	5.3
Injury/Accident	107	7.6
**Maternal Health**	**565**	**40**.**0**
Neurological	11	0.8
Non-Communicable	351	24.9
Respiratory	83	5.9
Others	55	3.9
Encounter period		
October 2021-September 2022	581	41.2
October 2022 – October 2023	830	58.8

### Teleconsultation outcomes

**Figure 2:**
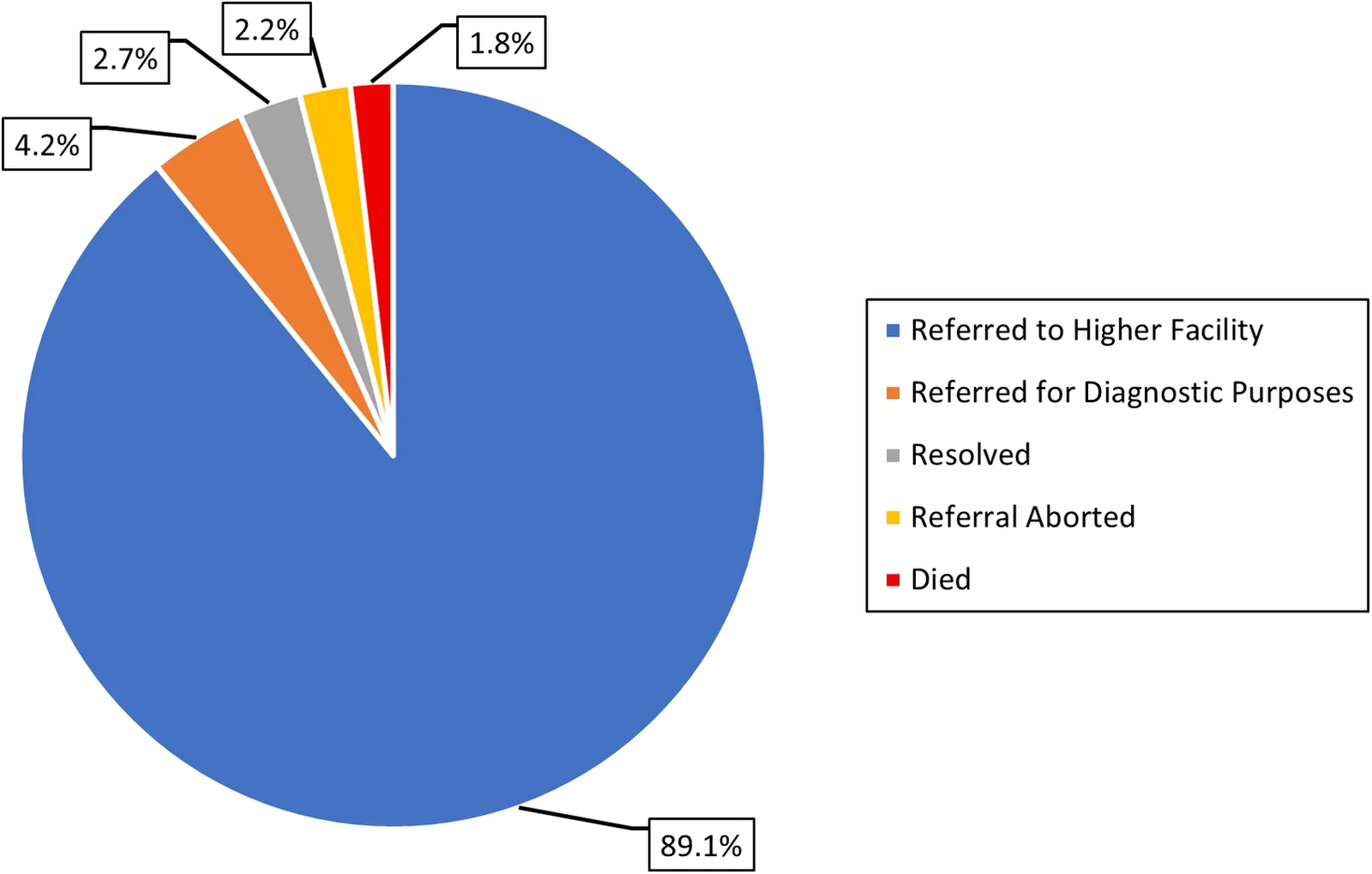
Overall tele consultation outcomes (*n* = 1,411).

 Figure 2 presents the overall outcomes of consultations over the two years. The majority of cases (89.1%) were referred to a higher facility for further management, 4.2% were referred for diagnostic purposes, 2.7% were resolved without onward referral, 2.2% had an aborted referral, and 1.8% resulted in death. Of the cases resulting in death, some occurred before referral could be initiated, while others occurred after referral to a higher-level facility.

### Teleconsultation outcomes by encounter period

**Figure 3:**
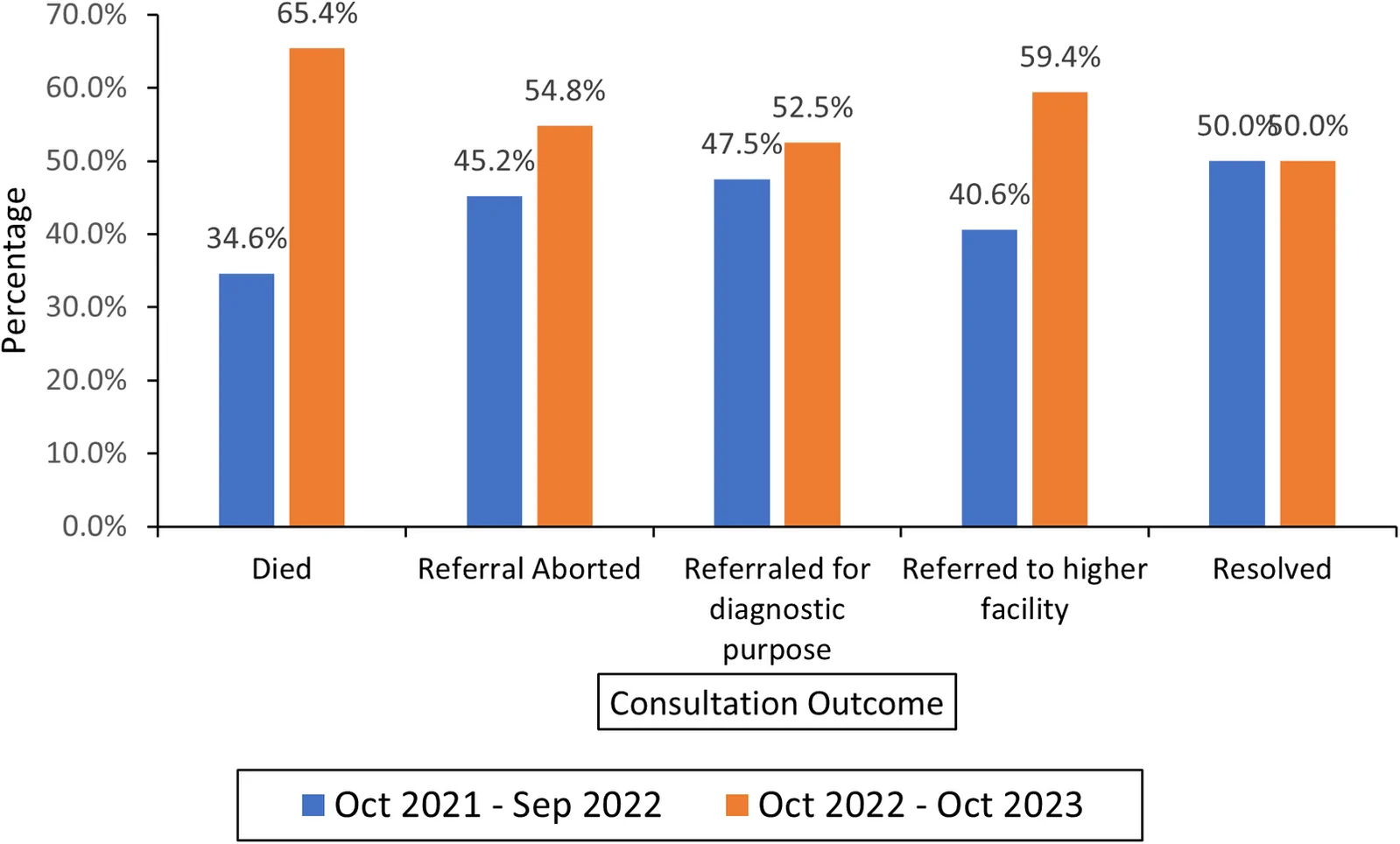
Distribution of telemedicine outcomes by encounter period (*n* = 1,411).

Figure 3 shows the distribution of Telemedicine Outcomes by Encounter Period. Of all teleconsultations that resulted in referral to a higher-level facility during the study period, 59.4% occurred between October 2022 and October 2023, compared with 40.6% between October 2021 and September 2022. Similarly, a greater share of deaths (65.4%) and aborted referrals (54.8%) were recorded in the latter period.

### Qualitative findings

#### Socio-demographic characteristics of health professionals

Table 2 presents the background characteristics of the health practitioners. The majority (56.5%) were aged between 30 and 39 years, while 26.1% were above 40 years. Most of the respondents were female (69.6%) and 60.9% had a diploma, whereas 39.1% held a degree. In terms of cadre, nurses constituted the largest proportion (52.2%), with physician assistants having the least representation (8.7%). The majority (73.9%) had worked in the health service for 1–9 years. Notably, 91.3% of the respondents had worked at the TCC for 2–3 years.

**Table 2:** Socio-demographic characteristics of health professionals (*n* = 23).

Variable	Frequency (*n* = 23)	Percent (%)
Age		
20–29	4	17.4
30–39	13	56.5
40 +	6	26.1
Sex		
Male	7	30.4
Female	16	69.6
Professional qualification		
Diploma	14	60.9
Degree	9	39.1
Cadre of Staff		
Midwife	9	39.1
Nurse	12	52.2
Physician Assistant	2	8.7
Years of working in the health service		
1–9 years	17	73.9
10–20 years	6	26.1
Years working at TCC (In years)		
<2	2	8.7
2–3	21	91.3

TCC, Teleconsultation Centre.

#### Socio-demographic characteristics of patients

Table 3 shows the socio-demographic characteristics of patients who participated in the in-depth interview. Most of the participants (56.3%) were females and the majority (37.6%) were above 60 years and about 25.0% were between 40 and 49 years. The largest proportion (25.0%) had no formal education, while 25.0% were traders. Most of the participants (62.5%) were also residents of Hohoe.

**Table 3:** Socio-demographic characteristics of patients (*n* = 16).

Variable	Frequency (*n* = 16)	Percentage (%)
Age		
20–29	2	12.5
30–39	2	12.5
40–49	4	25.0
50–59	2	12.5
60+	6	37.5
Sex		
Female	9	56.3
Male	7	43.8
Educational Level		
No formal education	4	25.0
Basic	1	6.3
Junior High School	3	18.8
Senior High School	2	12.5
Tertiary	6	37.5
Occupation		
Employed	6	37.5
Unemployed	3	18.8
Trader	4	25.0
Retired	3	18.8
Residence		
Hohoe	10	62.5
Jasikan	4	25.0
Likpe Mate	2	12.5

#### Thematic findings

Table 4 presents the themes from our analysis. These were roles of telemedicine, challenges and benefits of integrating telemedicine by healthcare professionals and patient satisfaction with the use of telemedicine.

**Table 4:** Thematic table from health professionals’ and patients interviews.

Main theme	Sub-theme
	• **Code**
Role of TCC	**Type of service**
	• Referral
	• Case management
	• Seek guidance (consultation from a specialist)
	**Access to healthcare**
	• Bridge geographical barrier
	• Saves cost for patient
	• Increase access to quality health care
	**Improvement in service delivery**
	• Pre-adequate referral management
	• Reduce waiting time
	• Reduce mortality (infant)
	• Confidence in providing service
	• Reduce risk of further complications
Challenges faced by health professionals	**Communication issues**
	• Network issues
	• Poor feedback from TCC
	• Unavailability of TCC at midnight
	**Logistical challenges**
	• Unavailable of telephones
	• Lack of a transport system
	• Lack of airtime
	• Lack of basic medical equipment
Patient Satisfaction	**Communication and service delivery**
	• Very satisfied
	• Satisfied
	**Patient challenges**
	• Network challenges
	• Language barrier

TCC, Teleconsultation Centre.

## Role of telemedicine in healthcare systems

Health professionals in our study expressed their perception of the role of telemedicine in the healthcare system. They focused on the type of services, access to healthcare, improvement of service delivery and quality of service. Telemedicine plays an important role in the provision of healthcare services. In general, telemedicine is used to provide healthcare services to people remotely and bridge the distance gap (11).

The types of services were referral, case management, and seeking guidance from a specialist. The health professionals stated that telemedicine has been instrumental in their field of work. It has enabled them to manage cases that were previously beyond their capacity and referral has become very easy for them. A midwife had this to say regarding the type of services.

“I remembered… it's not our client though from a different place, she was in labor. When she came, the HB was not good so we called to refer, and they said we should just go on with the delivery and then come to the hospital for blood. So I was there, and they brought the blood and then we transfused the woman, and everything was okay” (Midwife, female, 42 years).

“So most of the times we use the center to inquire or to seek for guidance and then for your referral process” (Midwife, Female, 34 years)

Telemedicine has increased accessibility to healthcare by bridging the geographical barrier, saving costs for patients and increased access to quality healthcare. They emphasized that distance is no longer a barrier when it comes to accessing healthcare. With the inception of telemedicine, they are able to provide service to patients regardless of their location. A midwife had this to say regarding accessibility

“When I came to this facility at first, when I had issues, I had to call the ward. Even in the ward, I have to call someone that I know. By the time they reach the doctor, the client will be in a worse situation. But with this TCC right now, things are flexible. We don't even have to stress ourselves more” (Midwife, Female, 40 years)

Improvement in service delivery in terms of pre-adequate referral management, reducing waiting time, confidence in providing service, and reducing the risk of further complications was also a key role of telemedicine. The health professional argued that with telemedicine receiving facilities in terms of referral are able to prepare adequately before the client arrives, thereby reducing delays and preventing potential complications. A nurse had this to say;

“So I can use one particular case, we had a client with a patient with difficulty breathing…. And when we got there, they were ready for us, their tubes were ready, they didn't ask us to go and get a folder first, because we had already provided them the information” (Nurse, Female, 28 years).

“Okay, there was a stomach ulcer, a severe one, which we were not able to diagnose early. But when I called, I got clarification on it, and then I was directed to give some medication, but if it still aggravates, then we refer to Hohoe and with that, I think it has helped a lot and the patient recovered without being referred” (Nurse, Male, 34 years)

### Challenges faced by health professionals

Telemedicine services encounter several challenges. Communication and logistical challenges were the main challenges reported by the health professionals.

Communication challenges such as network issues, poor feedback from TCC and unavailability of TCC at midnight were the main communication issues they encounter during service provision. Most of the health professionals reported that telemedicine in the Hohoe Municipality is primarily through phone calls and relies heavily on a stable network. However, network challenges have become a significant barrier to delivering these services effectively. A nurse had this to say

“Okay, the only challenge I have seen so far is network problems. Sometimes when we call, it is not from the teleconsultation team. It is a general problem. The network mostly didn't give us the access those times” (Nurse, Male, 34 years)

“So we have a lot of barriers, barrier number one is the network challenge, I mentioned the second barrier is about the road network” (Nurse, Female, 33 years)

Logistical challenges included the unavailability of telephones, transport systems, airtime, and basic medical equipment. They emphasized that the availability of essential equipment and resources plays a crucial role in the success of telemedicine services. These resources ensure the smooth operation of telemedicine, particularly access to airtime for phone consultations and ambulances for emergency transportation.

“Sometimes, if I refer a case now and I use my personal phone and you want to give feedback to the facility, sometimes it will be a problem. You have to call me again and all this, but if we have a dedicated phone for the facility…. Ah, then the facility will just receive the feedback” (Midwife, Female, 37 years)

“Okay, to my side, we have network challenges. Sometimes we do call our nurses, but it won't be going. And then transportation. Some cases need quick transport, like ambulances, but we don't have it” (Community Health Nurse, Female, 27 years).

### Patient challenges

Concerning the challenges from the patient's perspective, our study identified network and language barriers as challenges. However, most patients reported that there were no specific challenges that they encountered with telemedicine. They emphasized that telemedicine has rather improved the quality of health care they receive. A patient had this to say;

“There is an issue with the language; they mostly speak English instead of local languages” (Trader, Female, 35 years)

### Patient satisfaction with telemedicine

Patient satisfaction here was defined as the level of contentment of patients with the services that they receive from the health facility. Most patients had no issues with the health services provided by the health professionals. They generally have a positive experience with telemedicine services. They express satisfaction with how the service is conducted, particularly appreciating the reminders, follow-ups, and professional communication from the health professionals at the TCC. Here is a comment made by one of the patients;

“There is no problem at all. You see how you are doing it, it is good because someone might forget it is time for review and will not come but when you call, then they remember” (Trader, Female, 35 years)

Table 5 presents a joint display integrating the quantitative and qualitative findings

**Table 5:** Triangulation of findings.

Quantitative finding	Qualitative evidence	Type	Meta-inference
89.1% of encounters referred to a higher facility	Sub-themes “seek guidance” and “referral” (Role of TCC)	Convergence	Telemedicine functions primarily as a triage/referral-support bridge, not a stand-alone treatment endpoint
40.0% of encounters were maternal-health related	Illustrative account of a labor case managed via TCC	Convergence (illustrative)	Maternal care is the dominant, and clearly illustrated, clinical use-case in this system
Only 2.2% of referrals aborted	Patient-satisfaction theme (“very satisfied,” “satisfied”)	Convergence	Low referral abandonment aligns with reported patient satisfaction. The referral pathway is perceived as acceptable, without claiming a measured outcome effect
No quantitative measure of connectivity downtime was collected	"Network issues" sub-theme (Communication challenges)	Expansion (qualitative-only)	Qualitative data surface an operational barrier the routine records couldn't capture

TCC, Teleconsultation Centre.

## Discussion

In this study, we explored the role of telemedicine in bridging the healthcare gap using a mixed-method approach. The quantitative part assessed the current practices and standard routine of using telemedicine and the qualitative part explored the role of TCC in extending healthcare to remote areas, the challenges of telemedicine in rendering healthcare service, and patient health satisfaction with the use of telemedicine.

We found that four out of 10 telemedicine encounters (40.0%) were related to maternal health and almost nine out of 10 encounters ended up in referrals (89.1%). This highlights telemedicine's critical role in maternal and reproductive health (26). The predominance of female patients and maternal health cases in the telemedicine records reflects both the demographic profile of healthcare utilization in rural Ghana and the prioritization of maternal health services within the telemedicine program. Maternal health conditions often require timely specialist input, making them particularly suited to teleconsultation within a hub-and-spokes framework. The findings are consistent with Islam et al, who found that telemedicine increased maternity utilization and life-saving care (27). This finding can be attributed to the demand for women's healthcare, especially during pregnancy and the postpartum period. The outcome shows how well telemedicine works for women's health issues. However, it also highlights the need for ways to better engage male consumers, whose health-seeking behavior may be different or underrepresented (28). These findings align with our qualitative findings where healthcare professionals emphasize that telemedicine improved referral coordination and allowed them to manage complex cases with specialist guidance. For instance, the sub-theme “seek guidance” captured how lower-level providers consulted specialists at the TCC to clarify diagnoses and treatment plans. The alignment of the quantitative and qualitative findings shows telemedicine as a bridge between peripheral facilities and the main hospital, consistent with the HSM framework, which emphasizes the central role of the hub in supporting the spokes.

Furthermore, the high referral rate suggests that while telemedicine serves as a crucial first point of care, it often functions as a gateway to in-person services at higher-level facilities. This is consistent with the findings by Guille et al, which demonstrated that telemedicine-based maternal healthcare models successfully improved access and outcomes but still required in-person follow-ups for more complex cases (29). This could be attributed to a lack of healthcare facilities and specialists in LMIC and telemedicine bridges this gap by providing remote consultations (29). This finding implies that there is a limited local capacity, such as logistics and resources available at the spokes, resulting in the high referrals. However, telemedicine triages patients and ensures that those needing advanced treatment are sent to the right institutions for quality or improved health services. Barriers such as access to technology, infrastructure, and affordability must be addressed to ensure equitable healthcare delivery (30).

Our study also demonstrates that telemedicine plays an integral role in health service delivery across three key domains: accessibility, service improvement, and service type. Regarding accessibility, we found that telemedicine makes healthcare consistently available to individuals irrespective of their geographical location thus bridging the geographical barrier, saving cost for patient and increase access to quality health care. This finding corroborates existing studies (31–33), which highlights telemedicine's capacity to provide safe and convenient treatment options tailored to patients’ unique needs. This implies that telemedicine alleviates the strain on secondary healthcare facilities by enabling remote access to care.

Our findings also indicated that telemedicine is basically used for specialist guidance and referrals. Quantitatively, this was reflected in the high proportion of cases (89.1%) that required consultation or referral to the hub facility. Qualitatively, health professionals described telemedicine as a platform for seeking expert advice and support in managing cases beyond their capacity, as captured in the code “seek guidance.” This means that the quantitative result and health professionals’ experiences show how telemedicine strengthens the referral and support system. This aligns with a study by Asare et al, which emphasizes that telemedicine fosters peer-to-peer support among healthcare professionals and reduces hospital congestion (34). Furthermore, the study revealed a marked improvement in the quality of care since the implementation of telemedicine, a result consistent with research on its use in rural Ghana to mitigate medical professional shortages (35). The roles of telemedicine support our conceptual framework, Hub-and-Spoke (20, 22), where a central hub empowers remote facilities to deliver quality healthcare. By extending services to these areas, telemedicine not only enhances healthcare access and quality but also holds the potential to reduce mortality rates and health disparities in Ghana.

We also identified communication and logistical issues as primary threats to the efficacy of telemedicine services. With communication, inconsistent network connectivity and instances of one-way communication were found to impede the timely delivery of care. This is consistent with (35, 36), which stated that network challenges act as a critical hurdle for the adoption of telemedicine. On the logistical challenges, we found that a lack of reliable transportation, inadequate access to telephones, and insufficient funds for mobile airtime present major obstacles. These findings are consistent with Tchao et al. (35), who highlight that poor transportation hinders access to essential follow-up care, which can negate the benefits of remote consultations. Ghana faces a broader challenge of inadequate infrastructure for telemedicine deployment (35). These findings may be due to the geographical area of Hohoe Municipal, where the healthcare system faces several challenges, such as inadequate resources, inadequate emergency transport, poor road network, and the remote nature of most communities. This implies that the persistence of these barriers can compromise the timely and reliable delivery of healthcare, potentially undermining the very goals of telemedicine by reducing accessibility, diminishing service quality, and increasing the strain on higher-level health facilities.

Our study also found a positive experience (satisfied) with the use of telemedicine among the patients. This finding is in line with other studies (37–39). For instance, a study by Hamiel et al, emphasized that patients who use telemedicine, especially those in the paediatric demographic, have reported high satisfaction levels with genetic counselling that are similar to in-person sessions (40). However, while high levels of satisfaction are common, variations exist based on demographic factors and service dimensions. Mason et al. similarly found that sociodemographic factors such as gender, education level, and income were associated with variation in patient satisfaction, with women and those with lower education levels reporting comparatively higher satisfaction with the healthcare they received (41). The convergence of qualitative reports of patient satisfaction and the low proportion of aborted referrals (2.2%) quantitatively supports the effectiveness and acceptability of telemedicine in facilitating referrals. The positive experience reported by patients in this study suggests that telemedicine is an effective tool in delivering healthcare services, particularly in providing timely medical consultations and follow-ups. Healthcare institutions should consider expanding telemedicine services as a permanent option, particularly for follow-ups and non-urgent consultations. As patient-centered communication cannot be overstated, healthcare providers should receive training on virtual communication strategies to ensure that patients feel engaged and supported during teleconsultations (41).

Although this study was conducted in the Hohoe Municipality, the findings may apply to other districts in Ghana and similar rural settings across sub-Saharan Africa that share comparable healthcare system structures and infrastructural challenges. The Hub and Spokes Model used in this study provides a scalable framework that can be replicated in other regions to improve healthcare accessibility, strengthen referral systems, and promote capacity-building among lower-level health workers. The model also holds relevance for other LMICs facing health workforce shortages and uneven distribution of healthcare resources.

To accelerate the implementation and sustainability of telemedicine in Ghana and similar settings, several actionable steps are recommended. First, the Ministry of Health and Ghana Health Service should integrate telemedicine into the national health policy framework, ensuring dedicated funding and infrastructure support for rural facilities. Second, continuous digital literacy and capacity-building programs for healthcare workers are essential to enhance their confidence and proficiency in using telemedicine platforms. Third, improving network connectivity and providing facility-owned telecommunication devices and data packages will minimize logistical barriers and promote effective communication between hubs and spokes. Finally, partnerships between public and private stakeholders could help expand telemedicine coverage, strengthen referral networks, and ensure equitable access to care.

## Limitations

The qualitative sample was limited to 23 health professionals and 16 patients, which may not fully capture the perspectives of all stakeholders involved in telemedicine in the municipality. Again, telemedicine in our study was phone-based, which limits the ability to conduct physical examinations, visual assessments, and diagnostic evaluations. The quantitative component relied on a retrospective review of routine telemedicine records. Although efforts were made to include only complete and relevant records, the analysis was limited by the quality and scope of routinely collected data, which may not capture all clinical, operational, or patient-level factors influencing telemedicine outcomes. Additionally, the exact number of records excluded due to incompleteness was not documented during data extraction, so we were unable to assess whether excluded records differed systematically from those included; this represents an unquantified potential source of selection bias.

## Conclusion

The findings of this study emphasized telemedicine as a key strategy in advancing healthcare equity, which is the extension of healthcare services to hard-to-reach populations. The introduction of telemedicine in the Hohoe Municipality has demonstrated significant benefits in addressing healthcare inequalities as the Teleconsultation Centre (TCC) has improved the quality of healthcare delivery, improved health outcomes and increased accessibility to medical services, particularly in remote areas, through the application of the Hub and Spoke Model (HSM). This has also facilitated knowledge-sharing between healthcare professionals as specialists in the centre provide guidance and support to healthcare providers in rural areas, thereby strengthening the healthcare workforce and improving clinical decision-making. In addition, telemedicine has demonstrated the potential to enhance patient experience and continuity of care. These findings imply the need for integrating telemedicine into national and district-level health policies as a core component of referral systems. Also, there should be targeted investments in infrastructure at spoke facilities, such as diagnostic capacity, reliable connectivity, and essential medical equipment, which are necessary to reduce avoidable referrals and enhance case management. Overall, telemedicine serves as an instrument for achieving SDG 3 (3.1 and 3.8), which focuses on reducing maternal mortality and improving universal health coverage, respectively.

## Data Availability

The original contributions presented in the study are included in the article/Supplementary Material, further inquiries can be directed to the corresponding author.
