# Supplementary material for: Role of telemedicine in bridging the healthcare gap: application of the hub and spokes model in the Hohoe Municipality, Ghana
Source: Front Digit Health. 2026 Aug 12;8:1864794. doi: 10.3389/fdgth.2026.1864794 (PMC13508162; doi:10.3389/fdgth.2026.1864794)
Supplement: Supplementary file 1 [file Datasheet1.pdf]

Instrument

Appendix 1- INTERVIEW GUIDE FOR HEALTH PROFESSIONALS

Section A: Socio-demographic

What is your age? (*range*)

What is your sex?

What is your educational level

What is your professional qualification? (*Probe: certificate, diploma, degree, etc*)

What is your professional category? (*Probe: Are you a nurse, midwife, doctor, etc*)

How many years have you worked in the health sector?

How many years have you worked/used the Teleconsultation unit?

Section B: role of telemedicine in Extending Healthcare Services to Remote Areas

What types of healthcare services are commonly provided through teleconsultation? (*Probe: obstetrician services, mental health, pediatrics services)*

In your opinion, how effective is the Teleconsultation Centre in reaching and serving remote communities? (*Probe: Overcoming geographical barriers, increased accessibility, time-saving)*

How do you ensure the quality and accuracy of healthcare services delivered through teleconsultation? (*Probe: utilizing secure telecommunication platforms, having specialists (midwives, general nurses, mental health nurses, etc)*

Have you observed any improvements in healthcare access or health outcomes in remote communities since the implementation of teleconsultation services? (*Probe: Increased accessibility, reduced traveling burden for patients*

How will you rate patient satisfaction with telemedicine? *(Probe:* very h*igh, normal, low, dissatisfied)*

Section C: Challenges for the sustainable integration of Telemedicine in the healthcare system

Can you identify any challenges or barriers that hinder the integration of telemedicine into the healthcare system? (*Probe: network challenges, technological infrastructure, poor linkages between units)*

In your opinion, how can these problems be solved to improve services?

How do these challenges impact the effective delivery of healthcare services through telemedicine? (*Probe: slow delivery of services, limiting access, etc)*

APPENDIX 2: INTERVIEW GUIDE FOR PATIENTS

Section A: Socio-demographics

What is your age?

What is your sex?

What is your educational level? *(Probe: primary, JHS, SHS, tertiary)*

What is your employment status? *(Probe: self-employed, unemployed, government worker, etc)*

Where do you reside?

Section B: the impact of telemedicine on patient outcomes and satisfaction

Have you encountered any challenges or barriers when accessing healthcare through telemedicine? *(Probe: communication barriers, technical issues)*

How satisfied are you with the quality of care received through telemedicine? *(Probe:*

extremely satisfied with consultations, mixed feelings, dissatisfied)

In your opinion, how has telemedicine improved your health? *(Probe: significantly improved health, managing condition)*

APPENDIX 3- RECORDS REVIEW FORM

RECORDS REVIEW FORM FOR TELECONSULTATION		
**ENROLMENT DETAILS**		
Patient code	………………………..	
Date of Encounter	____/______/_________<br>(dd//mm//yyyy)	Time:
Condition	-------------------------------	
A cadre of staff consulted	………………	
**SOCIO-DEMOGRAPHICS OF PATIENTS**		
Age	……………..	
Sex	Male [ ]	Female [ ]
Name of facility	……………………………	
Community	……………………………….	

TREATMENT OUTCOMES		
Outcome	Resolved without transfer [ ]<br><br>Aborted (Referral aborted) [ ]	Referred to Volta Regional Hospital or other [ ]<br><br>Died [ ]
